# Investigation of the Factors Responsible for the Poor Oral Bioavailability of Acacetin in Rats: Physicochemical and Biopharmaceutical Aspects

**DOI:** 10.3390/pharmaceutics13020175

**Published:** 2021-01-28

**Authors:** Dong-Gyun Han, Eunju Cha, Jeongmin Joo, Ji Sun Hwang, Sanghyun Kim, Taeuk Park, Yoo-Seong Jeong, Han-Joo Maeng, Sang-Bum Kim, In-Soo Yoon

**Affiliations:** 1 Department of Manufacturing Pharmacy, College of Pharmacy, Pusan National University, Busan 46241, Korea; hann9607@pusan.ac.kr; 2 New Drug Development Center, Daegu–Gyeongbuk Medical Innovation Foundation, Daegu 41061, Korea; ejcha@dgmif.re.kr (E.C.); jjmin1422@naver.com (J.J.); hjs1228@dgmif.re.kr (J.S.H.); 3 Laboratory Animal Center, Daegu–Gyeongbuk Medical Innovation Foundation, Daegu 41061, Korea; shkim@dgmif.re.kr (S.K.); tw3000@dgmif.re.kr (T.P.); 4 College of Pharmacy and Research Institute of Pharmaceutical Sciences, Seoul National University, Seoul 08826, Korea; jus2401@snu.ac.kr; 5 Department of Pharmacy, College of Pharmacy, Gachon University, Incheon 21936, Korea; hjmaeng@gachon.ac.kr

**Keywords:** acacetin, solubility, stability, gastrointestinal absorption, tissue metabolism, bioavailability

## Abstract

Acacetin, an important ingredient of acacia honey and a component of several medicinal plants, exhibits therapeutic effects such as antioxidative, anticancer, anti-inflammatory, and anti-plasmodial activities. However, to date, studies reporting a systematic investigation of the in vivo fate of orally administered acacetin are limited. Moreover, the in vitro physicochemical and biopharmaceutical properties of acacetin in the gastrointestinal (GI) tract and their pharmacokinetic impacts remain unclear. Therefore, in this study, we aimed to systematically investigate the oral absorption and disposition of acacetin using relevant rat models. Acacetin exhibited poor solubility (≤119 ng/mL) and relatively low stability (27.5–62.0% remaining after 24 h) in pH 7 phosphate buffer and simulated GI fluids. A major portion (97.1%) of the initially injected acacetin dose remained unabsorbed in the jejunal segments, and the oral bioavailability of acacetin was very low at 2.34%. The systemic metabolism of acacetin occurred ubiquitously in various tissues (particularly in the liver, where it occurred most extensively), resulting in very high total plasma clearance of 199 ± 36 mL/min/kg. Collectively, the poor oral bioavailability of acacetin could be attributed mainly to its poor solubility and low GI luminal stability.

## 1. Introduction

Acacetin, an O-methylated flavone, is present in several medicinal plants, such as *Betula pendula* (silver birch), *Carthamus tinctorius* (safflower), *Robinia pseudoacacia* (black locust), and *Turnera diffusa* (damiana) [1,2,3]. Previous studies reported that acacetin exhibits several pharmacological effects such as antiperoxidative, anti-inflammatory, and antiplasmodial effects and possesses anticancer activities against breast, lung, prostate, and skin cancers [4,5,6,7,8]. Additionally, acacetin is the principal flavonoid in acacia honey, a very common and popular honey, produced from *R. pseudoacacia* flowers by bees [9]. This honey is known to have potential biological activities, including strong antioxidant, immunomodulatory, and antiproliferative properties, because of the presence of various bioactive constituents such as acacetin [10,11].

Despite their numerous health benefits, many flavonoids commonly exhibit very low oral bioavailability and insufficient levels in blood to produce therapeutic efficacy, which limits their successful clinical application [12]. Regarding mass balance, the oral bioavailability of a drug is determined by its stability in the gastrointestinal (GI) lumen, GI absorption, and intestinal/hepatic first-pass extraction [13]. Thus, the elucidation of the factors and mechanisms responsible for the poor oral bioavailability of a drug is a crucial prerequisite for the development of effective oral formulations [14]. For example, our group previously reported that limited intestinal absorption via the paracellular pathway was the primary mechanism underlying the poor oral bioavailability of doxorubicin [15]. This finding led to the successful development of a medium chain glycerides-based microemulsion system for the oral administration of doxorubicin [16]. Additionally, lovastatin is known to be efficiently metabolized in the liver [17], and this was the motivation for another study, in which proliposomes were developed as an oral delivery system that reduces the hepatic first-pass effects of lovastatin [18].

According to a recent systematic study on the in vitro and in vivo metabolism of acacetin in rats [19], the dominant phase I metabolism reaction of acacetin is oxidation, which is mainly catalyzed by cytochrome P450 (CYP) enzymes [19,20]. The major phase II metabolites of acacetin include monoglucuronide and monosulfate that are formed mainly by UDP-glucuronosyltransferase (UGT) 1A8 and sulfotransferase (SULT) 1A1, respectively [8,21]. In rats, 10 phase I metabolites, including apigenin, diosmetin, luteolin, and naringenin, and 21 phase II metabolites were identified in vivo [19]. Additionally, a study reported descriptive pharmacokinetic parameters such as terminal half-life (t_1/2_), peak plasma concentration (C_max_), and area under plasma concentration versus time curve from time zero to time infinity (AUC_inf_) following the intravenous dose of acacetin in rats [2]. However, to date, studies reporting a systematic investigation of the in vivo fate of orally administered acacetin are limited. Moreover, the in vitro physicochemical and biopharmaceutical properties of acacetin in the GI tract and their pharmacokinetic impacts remain unclear. This limited information regarding the oral absorption and disposition of acacetin may greatly hamper the rational design of oral formulations and their clinical applications.

Therefore, we aimed to elucidate the factors that determine the oral bioavailability of acacetin, such as its physicochemical properties, GI stability, GI absorption, hepatic first-pass extraction, and systemic clearance, based on the concepts of mass balance and a well-stirred hepatic clearance model. This study provides quantitative biopharmaceutical and pharmacokinetic insights to facilitate the design of efficient oral formulations and the precise prediction of herb–drug interactions for acacetin.

## 2. Materials and Methods

### 2.1. Materials and Animals

Acacetin (purity > 98%) and ketoconazole (KCZ) were purchased from Tokyo Chemical Industry Co. (Tokyo, Japan). Chlorpropamide (purity > 97%), quinidine (QND), sulfaphenazole (SPZ), α-naphthoflavone (NF), phosphate-buffered saline (PBS), dimethyl sulfoxide (DMSO), and polyethylene glycol 400 (PEG 400) were purchased from Sigma-Aldrich Co. (St. Louis, MO, USA). Nicotinamide adenine dinucleotide phosphate (NADPH), uridine diphosphate glucuronic acid (UDPGA), and rat liver microsomes (RLM; from male Sprague-Dawley rats) were purchased from Corning, Inc. (Midland, NC, USA). Pooled plasma (from male Sprague-Dawley rats) was purchased from Innovative Research, Inc. (Novi, MI, USA). The protocol for the animal studies conducted in this report was approved by the Institutional Animal Care and Use Committee at Pusan National University (approval no. PNU-2019-2217, date of approval: 17 April 2019). Eight-week-old male Sprague-Dawley rats weighing approximately 250 g were purchased from DBL Co. (Chungcheongbuk-do, Korea). The rats were housed and acclimatized before use, as reported previously [22].

### 2.2. Liquid Chromatography-Tandem Mass Spectrometry (LC-MS/MS) Method for the Determination of Acacetin

The concentration of acacetin in the samples obtained in this study was determined via a validated LC-MS/MS method using chlorpropamide as an internal standard (IS). The TSQ Vantage triple quadrupole mass spectrometer (Thermo Fisher Scientific Inc., Waltham, MA, USA) was coupled to an HPLC (Nexera XR; Shimadzu Co., Kyoto, Japan) with the TurboIon interface operated in positive ion mode using selected reaction monitoring. Chromatographic separation was performed using a reversed phase column (Kinetex C18, 2.1 mm × 100 mm, particle size 2.6 µm; Phenomenex, Torrance, CA, USA). Gradient elution of the mobile phase (consisting of 0.1% formic acid in water (solvent A) and acetonitrile (solvent B)) was performed at a flow rate of 0.3 mL/min, and the procedure was as follows (solvent A:solvent B, *v*/*v*): started at 80:20 at 0 min, ramped from 80:20 to 10:90 for 0.5 min, maintained at 10:90 for 0.5 min, back to 80:20 for 0.5 min, and maintained for 2 min (hence, the total run time: 3.5 min). The parameters related to the ion source were as follows: collision gas pressure, 1.5 mTorr; sheath gas pressure, 30 Arb; capillary temperature, 300 °C; vaporizer temperature, 300 °C; and auxiliary valve flow rate, 10 Arb. The method was validated according to the FDA guidelines [23], with regard to the accuracy, precision, selectivity, sensitivity, and linearity [24].

### 2.3. Determination of Solubility, Distribution Coefficients, and Permeability

Simulated gastric fluid (SGF) consisted of 0.2% (*w*/*v*) sodium chloride, 0.32% (*w*/*v*) pepsin, and 0.7% (*v*/*v*) HCl (final pH = 1.2). Simulated intestinal fluid (SIF) was prepared by dissolving 3 mM sodium taurocholate and 0.1% (*w*/*v*) pancreatin in phosphate buffer (final pH = 7.0). The solubility of acacetin was measured by adding excessive amounts of acacetin into glass vials containing 1.5 mL of water, pH 7.4 phosphate buffer, SGF, and SIF. The mixtures were allowed to reach equilibrium in a shaking incubator (FINEPCR, Gyeonggi-do, South Korea) at 500 rpm at 37 °C for 48 h. The samples were centrifuged at 16,000× *g* for 5 min, and the supernatant was passed through a 0.20-μm syringe filter to remove excess acacetin. Further, the concentration of acacetin in the filtrate was determined using the LC-MS/MS method. The distribution coefficients of acacetin in water (log P) and pH 7.4 phosphate buffer (log D_7.4_) were determined using an octanol–water system at 25 ± 0.5 °C. The two phases were pre-saturated with each other by vigorous vortex-mixing for 15 min and further stabilized overnight for phase separation. A certain amount of acacetin (less than its solubility) was dissolved in the pre-saturated octanol, vortex mixed with the same volume of pre-saturated water for 1 h, shaken for 24 h, and further centrifuged at 16,000× *g* for 30 min. Finally, the acacetin concentrations in the octanol and water layers were determined using the LC-MS/MS method. The membrane permeability of acacetin and reference compounds (verapamil as a reference for high permeability and ranitidine as a reference for low permeability) was assessed by parallel artificial membrane permeability assay (PAMPA). The PAMPA was conducted using a commercially available PAMPA kit (GIT PAMPA Double Sink; pION Inc., Massachusetts, MA, USA) according to the manufacturer’s protocol. The permeability was calculated using pION PAMPA Explorer ver. 3.8 software (pION Inc., Massachusetts, MA, USA).

### 2.4. Determination of Plasma Protein Binding and Blood Distribution

The fractions of unbound acacetin in plasma (fu_P_), microsomes (fu_MIC_), SGF (fu_SGF_), and SIF(fu_SIF_) were measured using a rapid equilibrium dialysis device (Thermo Fisher Scientific, Inc., Waltham, MA, USA) as previously described with slight modifications [25]. Briefly, 0.2 mL of the matrix spiked with acacetin (10 μM) was placed into the “sample” chamber, and 0.4 mL of PBS was placed into the adjacent “buffer” chamber. The fractions of unbound acacetin were calculated as the concentration ratio between the two chambers. The blood-to-plasma concentration ratio (R_B_) of acacetin was determined as described previously [25]. Briefly, 1-mL blood spiked with acacetin (1 μM) was incubated at 37 °C for 30 min. The blood sample was centrifuged at 2000× *g* for 5 min to prepare the plasma sample. The concentrations of acacetin in the aforementioned plasma and buffer samples were measured using the LC-MS/MS method.

### 2.5. Stability Study

The stability of acacetin was determined in several matrices such as phosphate buffers (pH 1.0, 3.0, 5.0, 7.0, 9.0, 11.0, and 13.0), plasma, RLM, SGF, and SIF. The samples spiked with acacetin (10 μM) were incubated at 37 °C, and 120 μL aliquots of the samples were collected at 0, 15, 30, 60, 120, 180, 240, 360, 420, 600, and 1440 min. The collected samples were diluted with acetonitrile to obtain a proper concentration and analyzed by the LC-MS/MS method.

### 2.6. Intravenous and Oral Pharmacokinetic Studies in Rats

The rats were fasted overnight before the experiment and anesthetized using an intramuscular injection of zoletil at 20 mg/kg [26]. The femoral artery and vein were cannulated with a polyethylene tube (Clay Adams, Parsippany, NJ, USA). Acacetin (dissolved in a vehicle composed of DMSO, ethanol, and PEG400 (10:5:85, *v*/*v*/*v*)) was administered intravenously (10 mg/kg) or orally (100 mg/kg) to rats. Approximately 250 μL of blood was collected via the femoral artery at 0, 2, 5, 15, 30, 45, 60, 120, and 180 min after intravenous administration and at 0, 2, 5, 15, 30, 45, 60, 90, 120, and 180 min after oral administration. The cannula was flushed with heparin (20 IU/mL in saline) at each time point to prevent blood clotting. Following centrifugation of the blood sample at 8000× *g* and 4 °C for 2 min, 120 μL of plasma was obtained. Urine was collected at 1-h intervals in an ice-cold container over 24 h. After urine collection at 24 h, each rat was sacrificed by cervical dislocation; the entire GI tract (including its contents and feces) was excised, transferred into a beaker containing 100 mL methanol, and cut into small pieces using scissors. After manual stirring with a glass rod for 1 min, a 50-μL aliquot of the supernatant was collected. The plasma, urine (Ae_24h_), and GI tract (GI_24h_) samples were stored at −80 °C until further LC-MS/MS analysis.

### 2.7. In Situ Closed-Loop Study

The in situ closed-loop study was performed as previously described with slight modification [15]. Briefly, after minimal abdominal incision under light ether anesthetization and sufficient washing of the contents within the GI tract, the jejunal loop was closed by ligation performed at approximately 2 cm distal to both ends of each jejunal section. Acacetin suspension (final concentration: 1 mg/mL) was prepared by dispersing acacetin powder in the vehicle solution for oral study and subsequently diluting it 5-fold with the SIF (final composition: 2% DMSO, 1% EtOH, and 17% PEG400 in the SIF). After injecting 0.2 mL of acacetin suspension into each loop using a l-mL 31-gauge syringe, the whole GI tract was carefully returned to its original position in the abdominal cavity. At 4 h after the injection, each loop was removed, transferred into a beaker containing 50 mL of methanol, and handled similar to the GI samples in the in vivo pharmacokinetic study. As control (in vitro isolated loop) groups, the jejunal closed-loop was prepared in the same manner as the in situ loop study, removed from the rat intestinal tract using scissors with special care not to damage the loop, and transferred to a glass beaker. Then, acacetin dispersed in the vehicle was injected into the isolated loop. After 0-h and 4-h incubation at 37 °C, the remaining fraction of acacetin in the isolated loop was measured in the same manner as the procedure for the GI sample in the in vivo pharmacokinetic study.

### 2.8. Metabolism Study in Rat Tissue S9 Fractions and RLM

The rat gut, spleen, kidney, liver, heart, brain, and lung were excised after cervical dislocation, rinsed with cold saline, blotted dry with tissue paper, and weighed. Each tissue sample was homogenized using a homogenizer (Ultra-Turrax T10 basi; IKA-Werke GmbH & Co. KG, Staufen, Germany) in cold 0.25 M sucrose solution. The homogenate was centrifuged at 9000× *g* for 30 min, and the supernatant fraction was stored at −80 °C until further use. The reaction mixture consisted of substrate, plasma, or tissue S9 fractions (protein concentration: 0.5 mg/mL) and NADPH (1 mM) or UDPGA (2 mM) in Tris buffer (100 mM; pH 7.4). After preincubation at 37 °C for 5 min, acacetin (final concentration: 10 μM) was added to initiate the enzyme reaction. After 5 min of incubation with shaking at 37 °C, the reaction was terminated by mixing the reaction mixture with acetonitrile containing IS (volume four times that of the reaction mixture). After vortex-mixing and centrifugation at 16,000× *g* for 10 min, 100 μL of the supernatant was obtained and stored at −80 °C until further LC-MS/MS assay.

To investigate hepatic phase I enzymes- and UGT-dependent metabolism of acacetin, the disappearance rate of acacetin at concentrations of 1–200 µM in RLM was measured in the presence of both NADPH and UDPGA. The substrate (acacetin) concentration ((S); µM) versus the initial metabolic rate (V; nmol/min/mg protein) profiles were interpreted based on Michaelis–Menten kinetics, and relevant parameters were estimated via nonlinear regression analysis (GraphPad Prism ver. 5.01; GraphPad Software, San Diego, CA, USA) using the following Michaelis–Menten equation:

$$V\;=\;\frac{V_{\max}\times \;[S]}{K_{m}\times \;[S]}$$

where K_m_ and V_max_ are the Michaelis–Menten constant and maximal metabolic rate, respectively. The unbound intrinsic metabolic clearance (CL_int_) was calculated as V_max_/(K_m_ × fu_MIC_). Furthermore, the disappearance of acacetin (20 μM) in the absence or presence of specific CYP isoform-selective inhibitors in RLM was determined to study the hepatic CYP-mediated metabolism of acacetin. Microsomal incubation mixtures (total volume of 200 μL) were prepared as follows: RLM (0.5 mg/mL microsomal protein), 1 mM NADPH, 10 mM MgCl_2_, 50 mM potassium phosphate buffer, acacetin, with or without CYP isoform-selective inhibitors (10 μM NF for rat CYP1A, 10-μM SPZ for rat CYP2C, 10-μM QND for rat CYP2D, and 10-μM KCZ for rat CYP3A). At 0 and 5 min after initiating the metabolic reaction, a 100-μL aliquot of the incubation mixture was taken, transferred into a clean 1.5 mL microcentrifuge tube containing 100 μL cold acetonitrile to terminate the metabolic reaction, and finally treated in the same manner as described for the tissue S9 fraction samples.

### 2.9. Pharmacokinetic Analysis

A noncompartmental analysis (WinNonlin ver. 3.1; Certara USA Inc., Princeton, NJ, USA) was performed to determine the following pharmacokinetic parameters: the AUC_inf_, area under plasma concentration versus time curve from time zero to the last sampling time (AUC_last_), t_1/2_, total body clearance (CL), apparent volume of distribution at steady state (V_ss_), and absolute oral bioavailability (F) [27]. The C_max_ and time to reach C_max_ (T_max_) were directly obtained from the plasma concentration versus time data.

### 2.10. Statistical Analysis

A *p*-value < 0.05 was considered statistically significant, calculated using a *t*-test to compare the means of two groups or Tukey’s honestly significant difference test with a posteriori analysis of variance (ANOVA) to compare the means of more than three groups. Unless indicated otherwise, all data are expressed as the mean ± standard deviation, except for median (ranges) for T_max_, and they are rounded to three significant digits.

## 3. Results

### 3.1. LC-MS/MS Method for Acacetin

**Figure 1:**
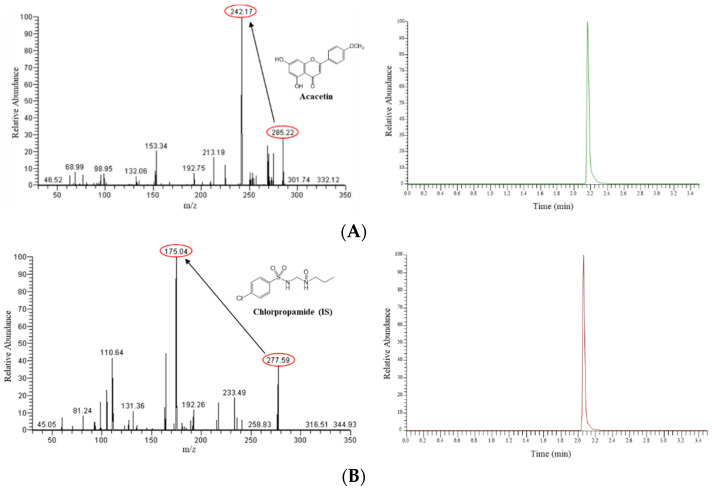
Representative product ion scan spectra (left side) and mass chromatograms (right side) of acacetin (**A**) and the internal standard (IS) (**B**) in rat plasma samples spiked with acacetin at high quality control level.

The product ion scan spectra and typical mass chromatogram for acacetin and the IS are shown in Figure 1. The most abundant precursor ions for acacetin and the IS were observed at *m*/*z* 285.22 and 277.59, respectively. The prominent product ions were detected at *m*/*z* 242.17 and 175.04, respectively. The selectivity of the LC-MS/MS method was assessed by comparing chromatograms from blank rat plasma. Isocratic elution using the reversed-phase column resulted in relatively short retention times of 2.2 min for acacetin and 2.1 min for the IS. No interference peak was detected around the retention times of the analyte in the blank plasma traces, indicating the good selectivity of the present method. For the evaluation of linearity, the calibration curves for acacetin were plotted with eight concentration levels, which were linear within the concentration range of 0.5–2000 ng/mL. The representative calibration curve for acacetin was *y* = 0.00314*x* + 0.253. The correlation coefficients of the four calibration curves were ≥0.996, indicating decent linearity. The lower limit of quantitation (LLOQ) value for acacetin was 0.5 ng/mL, which could offer sufficient sensitivity for the present study on rat pharmacokinetics. The within- and between-run precision was evaluated using triplicates of quality control samples at four concentration levels (LLOQ: 0.5 ng/mL; low quality control: 1.0 ng/mL; middle quality control: 100 ng/mL; high quality control: 500 ng/mL) on three different analytical runs (Table 1). The within-run accuracy for acacetin ranged from 93.8% to 101% with the precision ranging from 3.2% to 15.2%. The between-run accuracy ranged from 92.0% to 104% with the precision ranging from 0.9% to 14.6%. These validation results were within the acceptable range, indicating the accuracy and reproducibility of the established method [24,27].

### 3.2. Physicochemical Properties of Acacetin

The physicochemical properties of acacetin, such as lipophilicity, solubility, permeability, protein binding, and blood distribution were experimentally determined; the relevant parameters are listed in Table 2. The lipophilicity was assessed by measuring the partition (or distribution) of acacetin between n-octanol and water (or pH 7.4 phosphate buffer). Acacetin exhibited log P and log D_7.4_ values >3, indicating that it is a highly lipophilic compound [28,29]. Its solubility in water, pH 7.4 buffer, SGF, and SIF were ≤119 ng/mL, suggesting that acacetin can be considered a poorly soluble compound under biological conditions. The in vitro membrane permeability of acacetin was assessed using the PAMPA system. The rank order for the PAMPA permeability of the compounds tested was as follows: verapamil (81.4 × 10^−6^ cm/s) > acacetin (8.06 × 10^−6^ cm/s) > ranitidine (0.411 × 10^−6^ cm/s). The protein binding of acacetin was relatively extensive in plasma, moderate in RLM, and low in SGF/SIF. An R_B_ of approximately 1 indicated an equal distribution of acacetin between red blood cells and plasma.

### 3.3. In Vitro Stability of Acacetin

**Figure 2:**
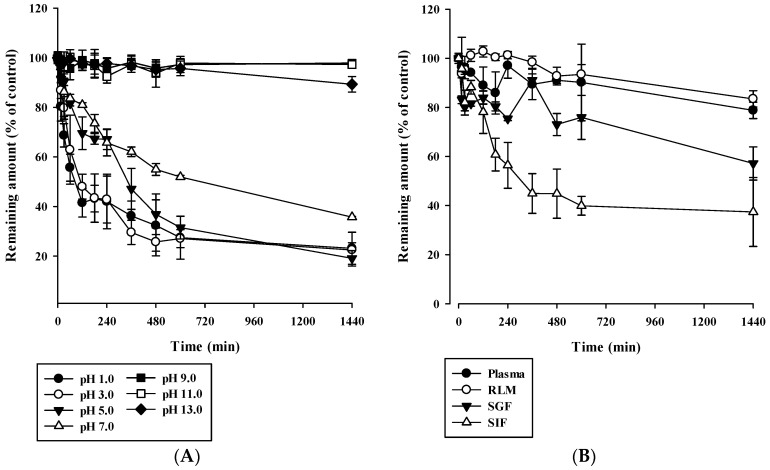
Stability profiles of acacetin in phosphate buffers of various pHs (**A**) and plasma, rat liver microsomes (RLM), SGF, and SIF (**B**). The bullet symbols and error bars represent the means and standard deviations, respectively (*n* = 3).

The stability profiles of acacetin in phosphate buffers of pH 1–13, plasma, RLM, SGF, and SIF are shown in Figure 2. It was regarded as stable when more than 85% of the initially spiked amount remained in the samples [23]. As shown in Figure 2A, acacetin was stable under basic conditions (pH 9, 11, 13), whereas it was unstable under acidic and neutral conditions; only 16.1–35.9% of the initially spiked acacetin amount remained after 24 h at the pH range of 1–7. As shown in Figure 2B, acacetin was stable in plasma and RLM (in the absence of NADPH and UDPGA/alamethicin), whereas it was unstable in SGF and SIF; 27.5–62.0% of the initially spiked acacetin amount remained in the samples after 24 h.

### 3.4. In Vivo Pharmacokinetics of Acacetin in Rats

**Figure 3:**
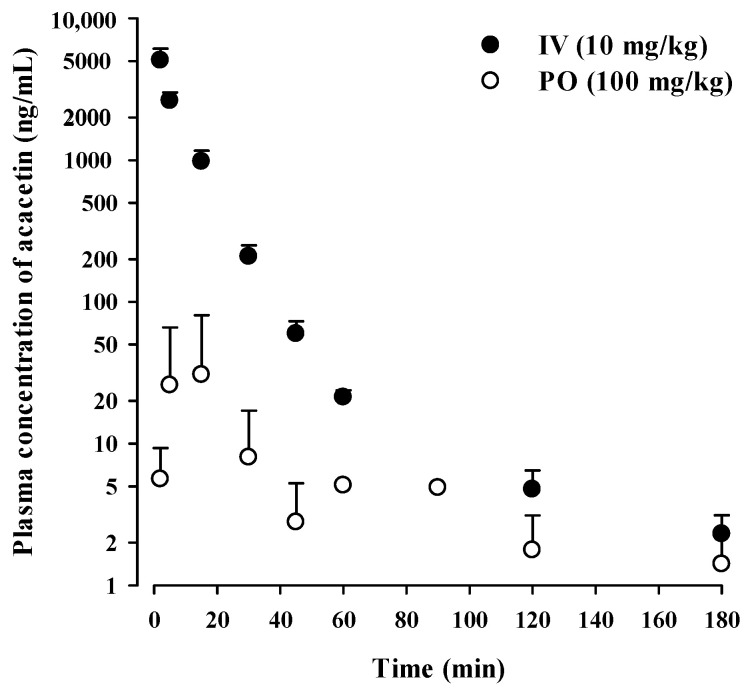
Plasma concentration versus time profiles of acacetin following intravenous administration at a dose of 10 mg/kg and oral administration at a dose 100 mg/kg in rats. The bullet symbols and error bars represent the means and standard deviations, respectively (*n* = 3–4).

The time profiles of plasma acacetin concentration following intravenous (10 mg/kg) and oral (100 mg/kg) administration in rats are shown in Figure 3, and the relevant pharmacokinetic parameters are listed in Table 3. Following intravenous administration, acacetin exhibited a very high CL with a relatively short t_1/2_. The urinary and GI (including biliary) excretion of unchanged acacetin contributed to minor fractions (1.5–12.8% of dose) of the CL of acacetin in rats. This indicates that a major fraction of acacetin administered intravenously is eliminated by metabolic processes. Following oral administration, considerable fluctuations and variations in plasma concentrations of acacetin were observed during the whole period of blood collection. Thus, the AUC_inf_, t_1/2_, and F of acacetin could not be estimated from the plasma concentration versus time curves, due to the absence of a discernible linear terminal phase. Assuming that acacetin follows linear pharmacokinetics with a constant renal clearance, the urinary excretion of unchanged drug is directly proportional to its plasma AUC_inf_ [30]. Thus, the F of acacetin can be estimated to be 2.34% using urinary excretion data.

### 3.5. In Situ GI Absorption of Acacetin in Rats

**Figure 4:**
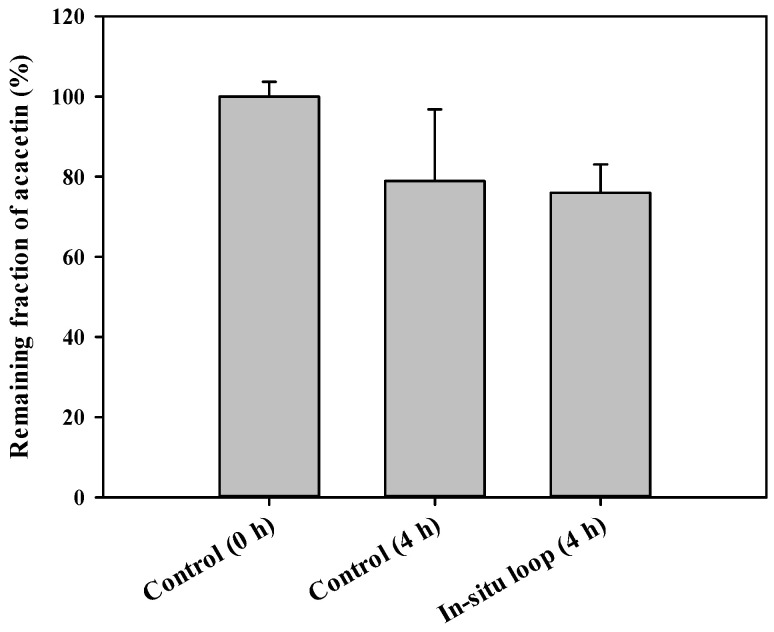
Remaining fractions of acacetin at 4 h after its injection into rat jejunum loops. The rectangular bars and error bars represent the means and standard deviations, respectively (*n* = 3).

The in situ absorption of acacetin in the GI tract was evaluated using a closed-loop assay. Figure 4 shows the remaining fractions of acacetin at 4 h after the injection of acacetin into the rat jejunal loops. The remaining fractions of acacetin ranged from 65.5% to 99.2% in the in vitro isolated loop and from 69.3% to 83.4% in the in situ loop after 4-h incubation. However, no significant differences in the remaining fractions were observed between the two groups (*p* = 0.806).

### 3.6. In Vitro Metabolism of Acacetin in Tissue S9 Fractions and RLM

**Figure 5:**
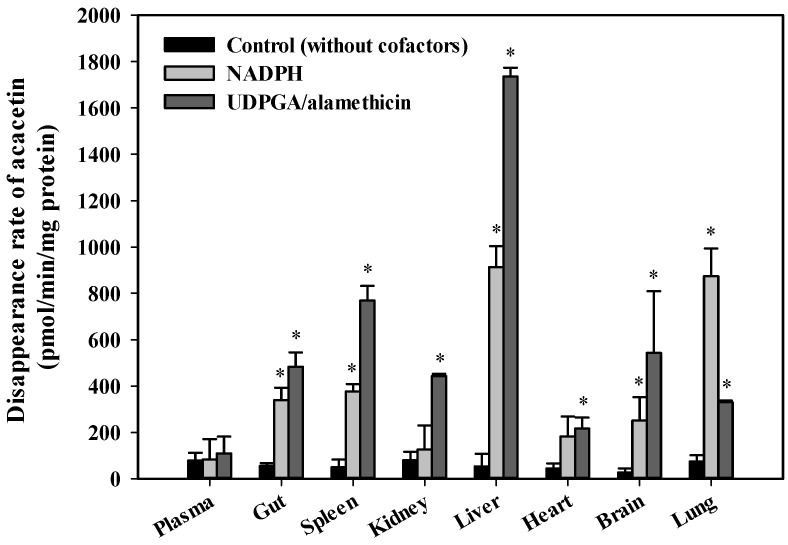
Disappearance rates of acacetin in rat plasma and various tissue S9 fractions in the presence of nicotinamide adenine dinucleotide phosphate (NADPH), (NADPH) and uridine diphosphate glucuronic acid (UDPGA)/alamethicin. The rectangular bars and error bars represent the means and standard deviations, respectively (*n* = 3). The asterisk indicates statistical significance when compared to the control group (* *p* < 0.05).

**Figure 6:**
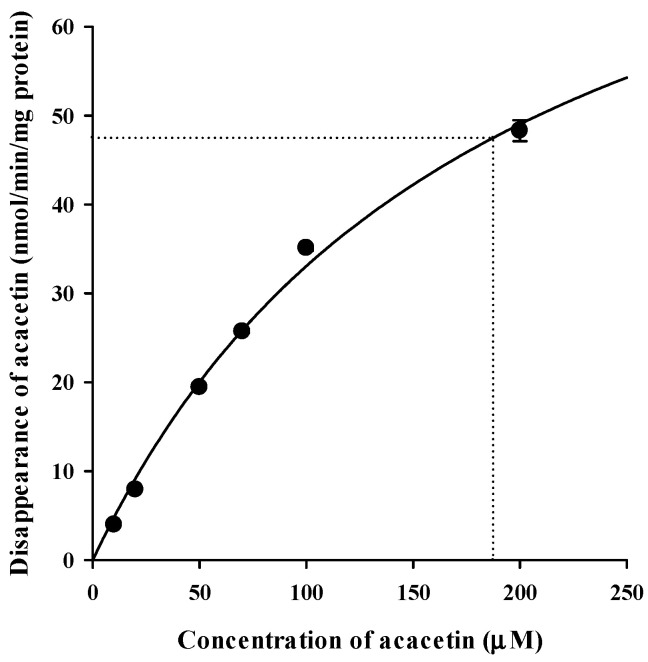
Concentration dependency for the disappearance of acacetin in RLM in the presence of NADPH and UDPGA/alamethicin. The bullet symbols and error bars represent the means and standard deviations, respectively (*n* = 3). The solid lines represent the fitted nonlinear regression curves.

**Figure 7:**
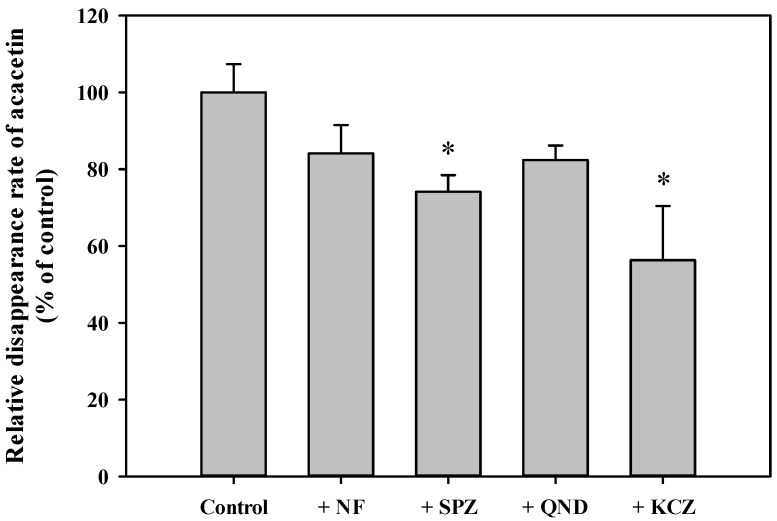
Relative disappearance of acacetin in RLM in the absence or presence of selective CYP inhibitors. The rectangular bars and error bars represent the means and standard deviations, respectively (*n* = 3). The asterisk indicates statistical significance when compared to the control group (* *p* < 0.05).

Tissue-dependent differences in the systemic metabolism of acacetin were assessed by measuring the disappearance rate of acacetin for 5 min in plasma and various tissue S9 fractions in the absence (control; without any cofactors) and presence of NADPH or UDPGA/alamethicin (Figure 5). In all tissues except the plasma, heart, and kidney, the disappearance rates were significantly higher in the presence of NADPH than in the control (*p* < 0.026). Similarly, in all tissues except the plasma, the disappearance rates were significantly higher in the presence of UDPGA/alamethicin than in the control (*p* < 0.034). Thus, the phase I enzymes- and UGT-mediated systemic metabolism of acacetin occurs significantly in most tissues. Notably, the liver exhibited significantly higher metabolizing activity than all the tested tissues; thus, the metabolic kinetics of acacetin was further investigated in RLM. The concentration dependency for the disappearance of acacetin in RLM in the presence of NADPH and UDPGA/alamethicin is shown in Figure 6. A saturable and concentration-dependent profile was well-described assuming the presence of one saturable component. The K_m_, V_max_, and CL_int_ were estimated to be 187 ± 14 μM, 95.0 ± 5.6 nmol/min/mg protein, and 508 ± 9 μL/min/mg protein, respectively. Although the phase I metabolism of acacetin is known to be mainly catalyzed by CYPs [19], the types of CYPs responsible for acacetin metabolism have not been identified so far. Thus, the disappearance of acacetin in RLM in the absence and presence of selective CYP inhibitors was further assessed. As shown in Figure 7, the relative disappearance rate of acacetin was significantly reduced in the presence of SPZ and KCZ by 25.8% and 43.7%, respectively (*p* = 0.021 and 0.001, respectively). This suggests that the CYP2C, 2D, and 3A subfamilies were involved in the metabolism of acacetin in RLM.

## 4. Discussion

The aim of this study was to investigate biopharmaceutical and pharmacokinetic factors responsible for the low oral bioavailability and high systemic clearance of acacetin. The pH-dependent stability test (Figure 2A) showed that acacetin was stable under basic condition (pH 9, 11, 13) but not stable under acidic and neutral condition (pH 1, 3, 5, 7). Moreover, as shown in Figure 2B, acacetin tended to be much more stable in the plasma (pH 7.4) and RLM (pH 7.4) than in the SGF (pH 1.2) and SIF (pH 7.0). These results could be attributed to other factors than pH conditions, because acacetin was observed to be unstable in acidic and neutral pHs (Figure 2A). Interestingly, the unbound fraction of acacetin was much lower in plasma and RLM (0.00901–0.317) than in SGF and SIF (0.707–0.849), as shown in Table 2. Thus, it is possible to speculate that a higher protein binding enhanced the stability of acacetin in the biological samples tested, which warrants further mechanistic studies on the effect of protein binding on the stability of phytochemicals including acacetin.

In the present rat pharmacokinetic study, the systemic exposure of acacetin following oral administration was observed to be very low and variable, resulting in a relatively low F of 2.34% and C_max_ of 52.1 ng/mL. A previous study reported that acacetin exhibited considerably high bioavailability of 84% and C_max_ of 1668 ng/mL following oral administration at 10 mg/kg [31]. However, considering that the bioavailability of flavonoids is generally low [12], this result seems to be fairly surprising and unusual. Moreover, contrary to the previous study, there have been other studies that reported a low systemic exposure of acacetin following oral administration as follows: F of 1.30% and C_max_ of 17 ng/mL at 5 mg/kg in mice [32] and C_max_ of 19.02 ng/mL at 1.4–1.676 mg/kg in rats [33]. Thus, the previously reported data on the bioavailability of acacetin were highly variable and controversial, but our present data clearly indicated a low oral bioavailability of acacetin in rats.

The PAMPA permeability values of acacetin seem to lie approximately in the middle of those of verapamil and ranitidine. This suggests that acacetin may have moderate membrane permeability via a passive diffusion pathway. Rats have been recognized as a good animal model for predicting the GI absorption of drugs, particularly via passive diffusion, in humans [15,34]. As a GI absorption prediction model, the maximum absorbable dose (MAD) can estimate a theoretical dose of a drug that can be absorbed across rat intestine, using solubility, intestinal volume, residence time, and intestinal permeability [35]. Based on the equilibrium solubility in the SIF measured in this study (119 ng/mL; Table 2) and Caco-2 cell permeability reported in a previous study (2.81 × 10^–6^ cm/s) [36], the MAD of acacetin can be estimated to be 3.61 μg/kg, which is a very small portion (0.00361%) of the orally administered dose (100 mg/kg). Theoretically, however, the estimate of MAD (0.00361%) which is much lower than the F (2.34%) does not make sense, suggesting that the MAD was under-estimated. As acacetin was poorly soluble and unstable in the aqueous media tested, its observed equilibrium solubility could have been under-estimated if the degradation rate is comparable to the dissolution rate during the present experimental procedures. Moreover, it is plausible that acacetin may be highly supersaturated in the dosing vehicle containing organic solvents, which can lead to a much higher transient concentration than the equilibrium solubility of acacetin in the SIF (119 ng/mL; classified as practically insoluble as per the United States Pharmacopeia (USP) criteria). Thus, the SIF solubility of acacetin measured in the present study could not represent an actual solubility of acacetin when it is dispersed in the dosing vehicle and subsequently administered into the GI fluids, which could result in the under-estimation of MAD. Alternatively, an in silico estimate of acacetin solubility in water can be used for the calculation of MAD. The predicted aqueous solubility values of acacetin ranges from 56 (determined by ALOGPS software; reported in the FooDB) to 650 μg/mL (determined by ACD software; reported in the SciFinder), which are classified as practically insoluble and very slightly soluble compounds, respectively, as per the USP criteria. Using these in silico solubility values, the MAD can be estimated as 1.7 and 19.7 mg/kg, respectively. This indicates that only 1.7–19.7% of orally-administered acacetin doses can be absorbable assuming the absence of degradation of acacetin in the GI lumen, which suggests that the limited GI absorption of acacetin may be mainly responsible for the low oral bioavailability of acacetin.

The fraction of a drug unabsorbed following oral administration can be estimated by comparing the GI_24h_ values between intravenous and oral pharmacokinetic studies. However, as this method is valid only when the drug is stable in the GI lumen for 24 h, it may be inadequate to use it for acacetin that has poor stability. Thus, it is difficult for us to estimate a reliable and accurate MAD or unabsorbed fraction of acacetin from the current data. In our present study, the GI absorption of acacetin was indirectly assessed by the in situ closed-loop study. It is important to note that both GI luminal degradation and GI absorption of drug occur simultaneously in the in situ loop state, whereas GI luminal degradation only occurs in the in vitro isolated loop state (control group). Thus, the real amounts of drug absorbed from the GI loop can be indirectly estimated by comparison of the remaining fraction observed in the in situ closed loop condition with that observed in the in vitro isolated loop condition. As shown in Figure 4, there were no significant differences in the remaining fraction of acacetin (*p* = 0.806) between the in vitro isolated loop and in situ loop groups (78.9% vs. 76.0%), suggesting that only a small portion (2.9%) of orally administered acacetin was absorbed from the loop. Assuming that these data hold true for the whole GI tract, the fraction of acacetin unabsorbed from the GI tract following oral administration can be estimated to be 97.1% of the dose (=100 − 2.9). Consequently, the fraction of acacetin dose eliminated by GI and hepatic first-pass metabolism before reaching the general circulation can be determined to be 0.56% of the dose (= 2.9 − 2.34). Thus, it can be speculated that the relative contribution of GI absorption and first-pass metabolism to the low oral bioavailability of acacetin is 97.1% and 0.56% of the dose, respectively. This suggests that the limited GI absorption caused by poor stability and solubility is mainly responsible for the low oral bioavailability of acacetin at the dose of 100 mg/kg. However, it appears that this result can be changed when either other dose levels vehicle systems, or both, are used, which warrants further investigation. Additionally, the total blood clearance (calculated as CL/R_B_) of acacetin is 191 ± 34 mL/min/kg, which far exceeds the hepatic blood flow rate of 50–80 mL/min/kg in rats [12,22]. As the liver exhibited the highest metabolic activity among various organs (Figure 5), it is speculated that acacetin is a drug with a high hepatic extraction ratio of nearly 1. Further systematic investigation of the mechanism of the hepatic disposition of acacetin is needed.

Collectively, the present results imply that the poor oral bioavailability of acacetin could be a consequence of the interplay among its poor solubility, low GI luminal stability, and intestinal permeability. Thus, formulation strategies to improve these unfavorable physicochemical properties could be effective for enhancing the oral bioavailability of acacetin [15,16,37]. It would be worthwhile to study various solubility/permeation enhancers and lipid-based nano-colloidal systems such as solid lipid nanoparticles, microemulsions, and nanostructured lipid carriers as potentially efficient oral delivery systems for acacetin.

## 5. Conclusions

The physicochemical compatibility, GI absorption, systemic metabolism, and pharmacokinetics of acacetin were comprehensively evaluated using relevant rat models. Acacetin exhibited poor solubility and relatively low stability in pH 7.4 phosphate buffer, SGF, and SIF. Moreover, the absorption of acacetin in rat jejunal segment was limited, and its F was very low at 2.34%. The systemic metabolism of acacetin occurred ubiquitously in various tissues (particularly in the liver, where it occurred most extensively), resulting in very high CL. Thus, the poor oral bioavailability of acacetin could be attributed mainly to its poor solubility and low GI luminal stability. To our knowledge, this is the first systematic study on the biopharmaceutical and pharmacokinetic factors responsible for the oral absorption and systemic disposition of acacetin.

## Figures and Tables

**Table 1:** Within-run and between-run precision and accuracy of the present bioanalytical method for the quantification of acacetin in rat plasma (*n* = 3).

Nominal Concentration (ng/mL)	Precision (%)		Accuracy (%)	
	Intra-Day	Inter-Day	Intra-Day	Inter-Day
LLOQ (0.5)	15.2	14.6	97.5	104
LQC (1)	12.0	9.0	101	93.9
MQC (100)	4.8	4.1	93.8	92.0
HQC (500)	3.2	0.9	99.1	101

**Table 2:** Physicochemical properties of acacetin (*n* = 3).

Property	Parameter	Value
Lipophilicity	Log P	3.51 ± 0.09
	Log D_7.4_	3.40 ± 0.20
Solubility (ng/mL)	Water	40.1 ± 7.3
	pH 7.4 phosphate buffer	12.1 ± 1.1
	SGF	59.3 ± 7.1
	SIF	119 ± 8
Permeability (×10^−6^ cm/s)	PAMPA	8.06 ± 0.91
Distribution	fu_P_	0.0102 ± 0.0011
	fu_MIC_	0.302 ± 0.021
	fu_SGF_	0.822 ± 0.026
	fu_SIF_	0.714 ± 0.009
	R_B_	1.04 ± 0.10

**Table 3:** Pharmacokinetic parameters of acacetin following intravenous administration at a dose of 10 mg/kg and oral administration at a dose 100 mg/kg in rats (*n* = 3–4).

Parameter	IV 10 mg/kg	PO 100 mg/kg
AUC_inf_ (μg∙min/mL)	51.3 ± 9.2	ND ^b^
AUC_last_ (μg∙min/mL)		0.771 ± 0.415
V_ss_ (mL/kg)	2220 ± 507	
CL (mL/min/kg)	199 ± 36	
t_1/2_ (min)	40.3 ± 7.2	ND ^b^
T_max_ (min)		5 (2–15)
C_max_ (ng/mL)		52.1 ± 50.5
Ae_24h_ (% of dose)	2.50 ± 1.15	0.0601 ± 0.0634
GI_24h_ (% of dose)	6.66 ± 5.34	29.3 ± 22.3
F (%) ^a^		2.34

^a^ Calculated as the dose-normalized amount of acacetin excreted in the urine for 24 h after oral administration divided by that after intravenous administration. ^b^ Not determined.

## Data Availability

The data presented in this study are available in the paper.
